# A New Prognostic Risk Signature of Eight Ferroptosis-Related Genes in the Clear Cell Renal Cell Carcinoma

**DOI:** 10.3389/fonc.2021.700084

**Published:** 2021-06-25

**Authors:** Ji Chen, Yating Zhan, Rongrong Zhang, Bo Chen, Junting Huang, Chunxue Li, Wenjie Zhang, Yajing Wang, Yuxiang Gao, Jianjian Zheng, Yeping Li

**Affiliations:** ^1^ Key Laboratory of Diagnosis and Treatment of Severe Hepato-Pancreatic Diseases of Zhejiang Province, The First Affiliated Hospital of Wenzhou Medical University, Wenzhou, China; ^2^ Department of Urology, The First Affiliated Hospital of Wenzhou Medical University, Wenzhou, China

**Keywords:** TCGA database, ferroptosis, ccRCC, prognosis signature, immune infiltration

## Abstract

Clear cell renal cell carcinoma (ccRCC) is the most common renal cell carcinoma and has poor prognosis in the locally advanced stage. Ferroptosis, a relatively new type of cell death, has gained significant attention in recent years. This study aimed to explore the prognostic value of ferroptosis-related genes (FRGs) in ccRCC. In this study, 50 differentially expressed FRGs between ccRCC and adjacent normal kidney tissues were identified, 26 of them correlated with overall survival (OS) (*P <*0.05). Eight optimal FRGs were selected by Lasso regression and multivariate Cox regression analysis, and used to construct a new prognostic risk signature to predict the prognosis of ccRCC patients. In addition, the signature passed the validation of prognostic survival analyses by a significant margin, and the risk score was identified as an independent prognostic marker *via* Cox regression analyses. Further studies indicated that the signature was significantly correlated with immune cell infiltration. Moreover, the levels of eight FRGs were examined in ccRCC. Collectively, the 8-FRG prognostic risk signature helps the clinicians predict the prognosis and OS of the patients, and standardize prognostic assessments.

## Introduction

Renal cancer accounts for approximately 2–3% of the adult malignancies and 80–90% of the adult renal malignancies (1). Approximately 80% of the RCC cases are Clear cell renal cell carcinoma (ccRCC) (2). Often asymptomatic in the early stages, ccRCC is suspected when the tumor volume increases and the patient develops fever, fatigue and other systemic symptoms (3). In addition, the microscopic appearance is often confused with granular cell carcinoma and spindle cell carcinoma, which makes it very difficult to grade under microscope (4). Recent studies have shown that high-risk ccRCC patients treated with the active drugs have no significant changes in the overall survival (OS) (5). To monitor the disease progression, the scientific community should explore novel and effective biomarkers for ccRCC prognosis, including the new prognostic signatures.

Ferroptosis is a novel cell death modality that has recently been investigated (6). With the advent of malignant drug-resistant tumors and the weakening of the effect of conventional anticancer treatment, the induction of ferroptosis in cells has become a new promising treatment for various cancers (7, 8). Increasing evidence has demonstrated that ferroptosis plays a key role in the regulation of the progression of various human cancers, including Head and neck squamous cell carcinoma (9, 10). CISD1, a typical ferroptosis-related gene (FRG), negatively regulates ferroptosis (11). In contrast, NCOA4 and MT1G have been found to sustain ferroptosis (12, 13). However, the roles of FRGs in the prognosis of ccRCC remain largely unknown.

In this study, we screened eight optimal FRGs to construct a new prognostic risk signature according to transcriptional and relevant clinical data of ccRCC patients obtained from the TCGA database. The prognostic value of this signature was verified *via* a series of OS-related analyses. In addition, the clinical traits and immune mechanisms of this prognostic risk model were analyzed to validate the accuracy of the signature. Finally, the levels of eight FRGs from the signature were examined in 20 paired ccRCC tissues and adjacent non-tumorous tissues by quantitative real-time PCR (qRT-PCR).

## Materials and Methods

### Database

All the mRNA expression files were obtained from the TCGA portal using the GDC tool (https://portal.gdc.cancer.gov/). The files contained data about ccRCC (n = 539) and adjacent nontumorous kidney samples (n = 72). Corresponding clinicopathological characteristics, consisting of OS and cancer specific survival (CSS) for ccRCC patients (n = 533), were also obtained from the TCGA database.

According to the patients’ ID numbers, we matched their transcriptomic data and clinical information, the data of the mismatched patients were removed. Thus, we obtained complete gene expression profiles of 526 ccRCC patients. Using R package “caret”, all ccRCC patients were randomized into two cohorts: the training cohort and the testing cohort (7:3). Specific clinical parameters for the two cohorts and the entire TCGA cohort were shown in **Table 1**. A total of 60 FRGs utilized in this study were obtained from the previous literature (**Supplementary Table 1**) (7). The 318 transcription factors (TFs) and relevant contents of immune cells of the TCGA database were downloaded from CISTROME (http://cistrome.org/) (14).

**Table 1:** The clinical characteristics and associated cohorts of 526 ccRCC patients.

Clinical parameters	Variable	Entire TCGA cohort (n = 526)	Training cohort (n = 371)	Testing cohort (n = 155)
Age (year)	>65	179	130	49
	≤65	347	241	106
Gender	female	185	130	55
	male	341	241	100
Grade	G1	12	10	2
	G2	227	162	65
	G3	205	140	65
	G4	75	54	21
	GX	7	5	2
Stage T	T1	267	183	84
	T2	69	51	18
	T3	179	127	52
	T4	11	10	1
Stage N	N0	238	167	71
	N1	16	11	5
	NX	272	193	79
Stage M	M0	418	301	117
	M1	78	50	28
	MX	30	20	10
Treatment_type	Radiation Therapy, NOS	262	181	81
	Pharmaceutical Therapy, NOS	264	190	74

### Identification of Prognostic Differentially Expressed Ferroptosis-Related Genes

The “limma” R package was performed to measure the differential expression in the training cohort, the false discovery rate (FDR) was calculated by the Benjamin–Hochberg method (15). In brief, the prognostic differentially expressed ferroptosis-related genes (PDEFRGs) were identified *via* univariate Cox analysis, only DEFRGs with FDR less than 0.05 were identified as OS-related genes. In this study, FRGs significantly associated with OS were considered as prognosis related FRGs. In addition, the Venn diagram was drawn to show these genes.

### Protein–Protein Interaction (PPI) Network of PDEFRGs

To explore the PPI relationships between PDEFRGs, a PPI network was performed by the STRING database (version 11.0) and Cytoscape software 3.6.1 (https://cytoscape.org/) (16). In addition, the connections between TFs and FRGs were determined by Cytoscape software.

### Generation of the 8-FRG Prognostic Risk Signature

We removed those FRGs that were over fit to the model *via* least absolute shrinkage and selection operator (Lasso) regression analysis (17). Eight optimal FRGs were finally selected by the multivariate Cox regression analysis and their regression coefficients were calculated (18). The regression coefficients and the expression levels of eight FRGs were used to achieve the risk score of each ccRCC patient, based on the following formula:

Risk score=Σ (expression level of gene×coefficient)

According to the cut-off value, which was the median risk score of the training cohort, we categorized the ccRCC patients in each cohort into two groups: high-risk and low-risk groups. Thus, the 8-FRG prognostic risk signature was generated from the training cohort.

### Survival and Immune Analyses

Kaplan–Meier curves and the operating characteristic curve (ROC) analysis were created to calculate the prognostic value of the 8-FRG prognostic risk signature. For ROC analysis, an area under the ROC (AUC) value >0.70 means that the model has an excellent predictive value (19, 20). Using the univariate and multivariate Cox regression analyses, several essential clinical characteristics and the 8-FRG prognostic risk signature were further analyzed. Next, the independent prognostic factors of ccRCC were included into the FRGs-clinical nomogram. The calibrate curve analysis and decision curve analysis (DCA) were applied to validate the accuracy of the nomogram. Finally, the single-sample gene set enrichment analysis (ssGSEA) was performed to obtain the infiltrating score between high- and low-risk groups (21). The relevant gene set file of GSEA analysis used in ssGSEA is provided in **Supplementary Table 2**. Utilizing the relevant contents of six main immune cells of TCGA database, the immune correlation analysis was performed *via* R package “corrplot”.

### Validation of qRT-PCR

We obtained 20 pairs of ccRCC and adjacent tumor tissue samples from The First Affiliated Hospital of Wenzhou Medical University. The use of these clinical samples was approved by the ethics committee of The First Affiliated Hospital of Wenzhou Medical University. For this study, patients signed a written informed consent. qRT-PCR was performed to evaluate the differences in the mRNA expression. The total RNA from ccRCC and adjacent normal tissues was extracted using TRIzol reagent. The mRNA was then reverse transcribed into cDNA using ribo SCRIPTTM reverse transcription kit. The expression level of mRNA was calibrated with glyceraldehyde-3-phosphate dehydrogenase (GAPDH). SYBR Green master mix was added, and real-time PCR was carried out using a 7500 rapid quantitative PCR system (Applied Biosystems, USA). The CT value of each well was recorded, and the relative quantification of the amplified products was performed using the 2^−ΔCt^ method.

### Statistical Analysis

The R software (version 4.0.2) downloaded from (https://www.r-project.org/) was utilized to perform all statistical analyses. The rank correlation was further assessed through the performance of the Pearson correlation coefficient test among the different variables. Independent t-tests were also performed to compare gene expression among different tissues. In all analyses, we set the statistical significance at *P <*0.05.

## Results

### Twenty-Six Prognostic Differentially Expressed Ferroptosis-Related Genes Were Identified

**Figure 1:**
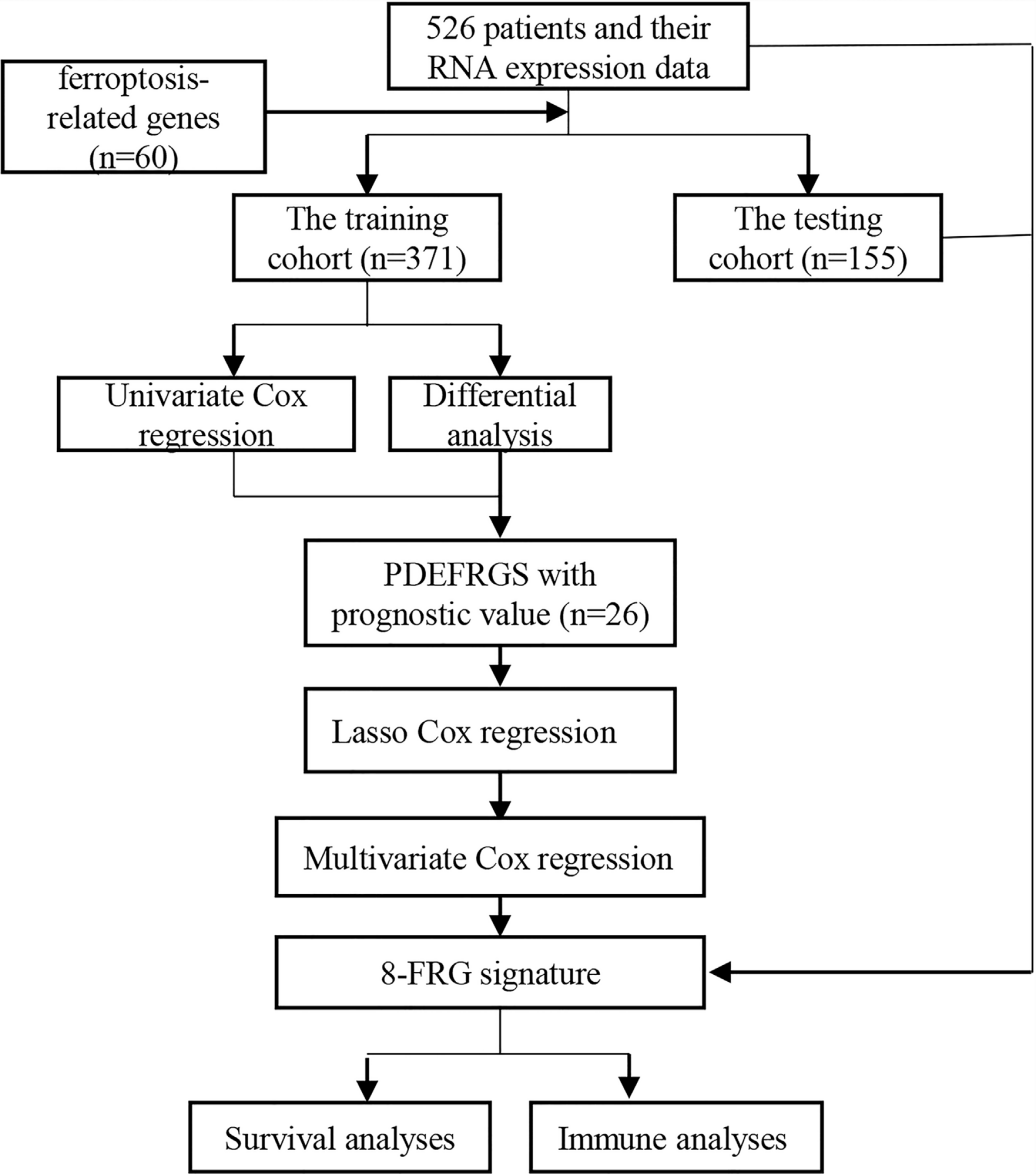
The overall workflow of this study.

**Figure 2:**
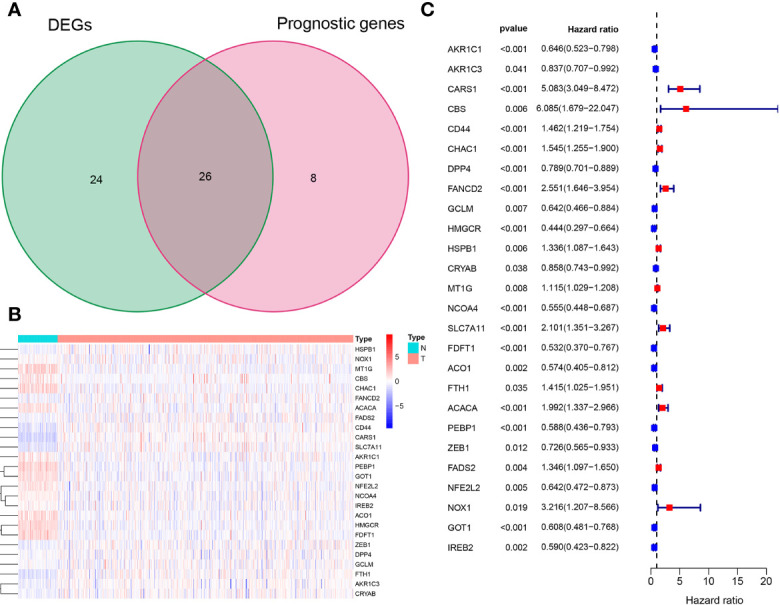
Identification of 26 PDEFRGs. **(A)** Venn diagram showing 26 PDEFRGs between DEFRGs and prognostic genes. **(B)** The heat map of 26 PDEFRGs. **(C)** Forest plots showing that 26 PDEFRGs correlated with OS (*P* < 0.05).

**Figure 3:**
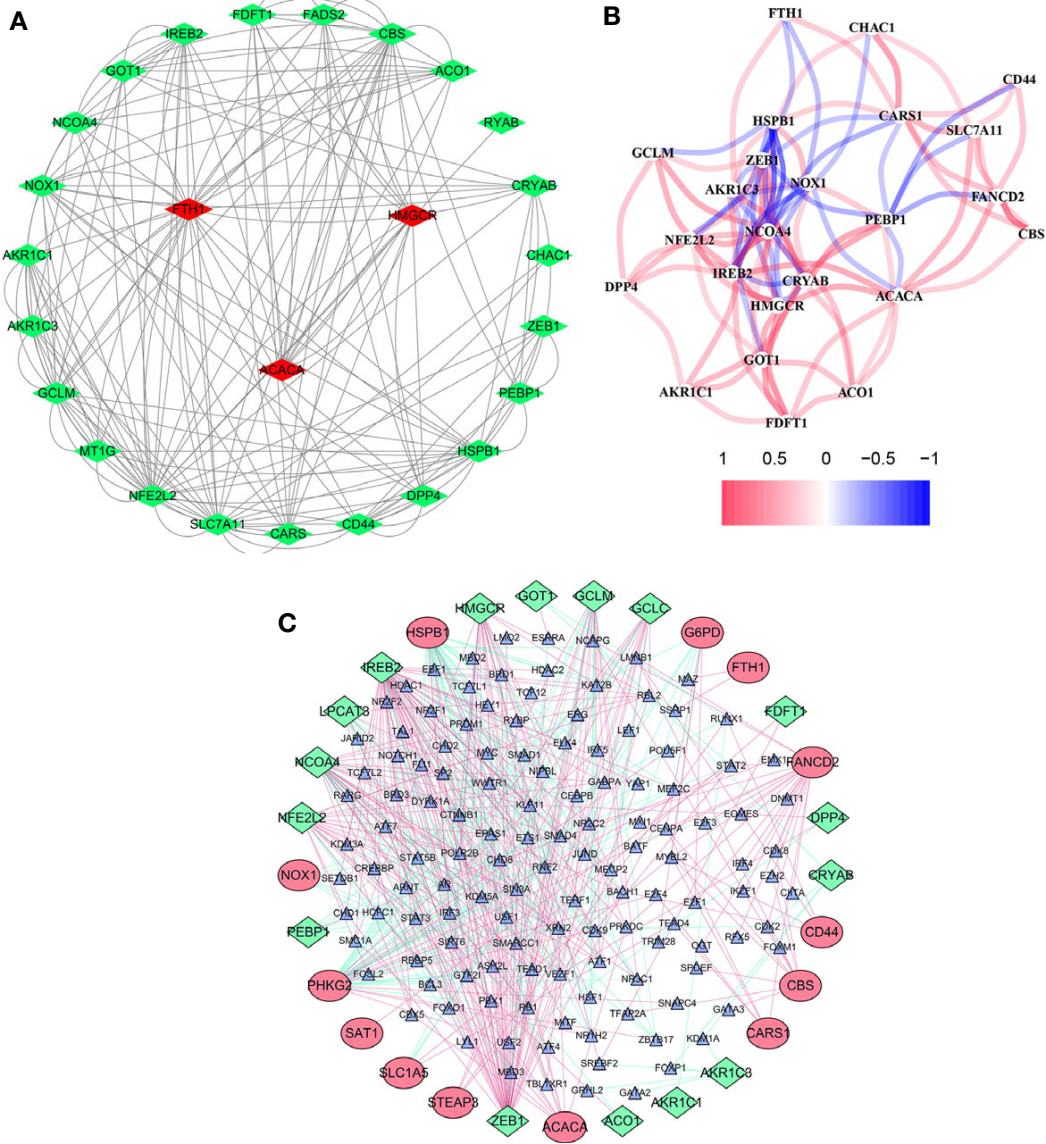
Network of FRGs. **(A)** PPI network constructed with the nodes with interaction confidence value >0.15 of 26 PDEFRGs. **(B)** The correlation network of 26 PDEFRGs. Different colors represent the correlation coefficients. **(C)** TFs-FRGs network; the green nodes: FRGs with low risk (*P* < 0.05), the red nodes: FRGs with high risk (*P* < 0.05), the blue nodes: TFs that correlated with the FRGs (correlation coefficient >0.4).

The overall workflow of this study is shown in **Figure 1**. In the training cohort, most of the FRGs (50/60, 83.3%) were differentially expressed in ccRCC tissues as compared with adjacent non-tumorous tissues. Via univariate Cox regression analysis, we identified 26 of them were significantly correlated with OS (*P <*0.05). Thus, 26 PDEFRGs were selected, as shown in Venn diagram (**Figures 2A–C**). Through the PPI network, we found that ACACA, FTH1 and HMGCR may be the hub genes (**Figure 3A**). The correlation of these PDEFRGs was shown in **Figure 3B**. Among 318 TFs, 253 were found significantly associated with differential expression of all FRGs. Thus, we developed a TFs-FRGs regulatory network to explain the regulatory relationships extensively (**Figure 3C**).

### Eight Optimal Prognostic Differentially Expressed Ferroptosis-Related Genes Were Selected in the Training Cohort

**Figure 4:**
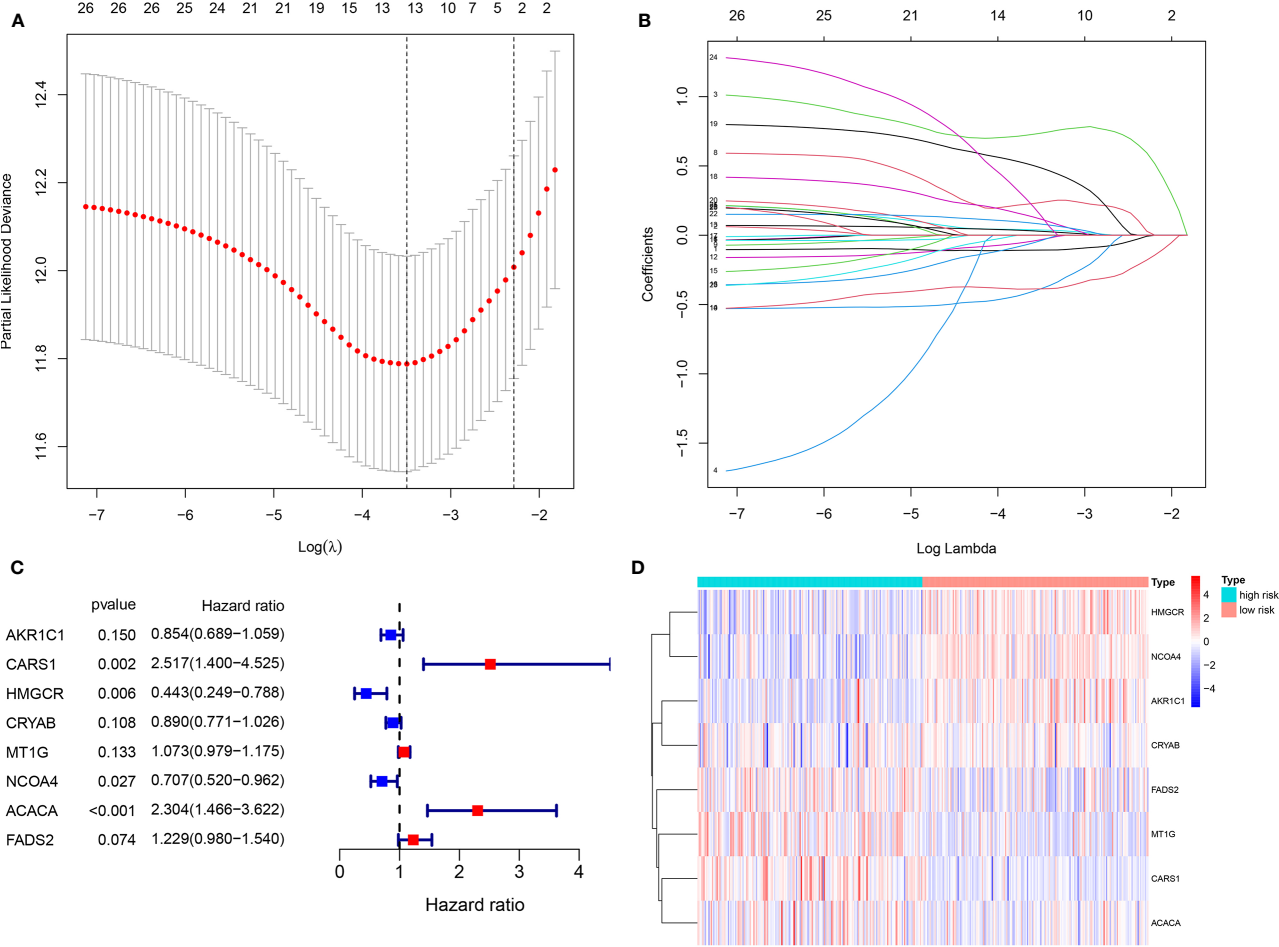
Identification of eight optimal FRGs. **(A, B)** LASSO regression analyses of 26 PDEFRGs. **(C)** Forest plots showing eight selected optimal risk FRGs. **(D)** The heat map of eight optimal FRGs.

Using the Lasso regression analysis, we removed 15 PDEFRGs that were overfit to the model (**Figures 4A, B**). Then, the multivariate Cox regression analysis was used to select eight optimal FRGs: AKR1C1, CARS1, HMGCR, CRYAB, MT1G, NCOA4, ACACA and FADS2 (**Figures 4C, D**). Among them, CARS1, MT1G, ACACA and FADS2 were identified as high-risk genes while AKR1C1, HMGCR, CRYAB and NCOA4 were categorized as low-risk genes. Moreover, the coefficients of eight FRGs were obtained through multivariate Cox regression analysis.

### Generation of the Eight Ferroptosis-Related Genes Prognostic Risk Signature

The mRNA expression levels and relevant coefficients of the eight optimal PDEFRGs were used to calculate the risk score as per the following formula:

$$\begin{array}{c} \text{Risk score}=\;(-0.1579\times \text{AKR}1\text{C}1)+(0.9231\times \text{CARS}1)+(-0.8143\times \text{HMGCR})+(-0.1171\times \text{CRYAB})\\ +\;(0.0701\times \text{MT}1\text{G})+(-0.3461\times \text{NCOA}4)+(0.8348\times \text{ACACA})+(0.2060\times \text{FADS}2). \end{array}$$

**Figure 5:**
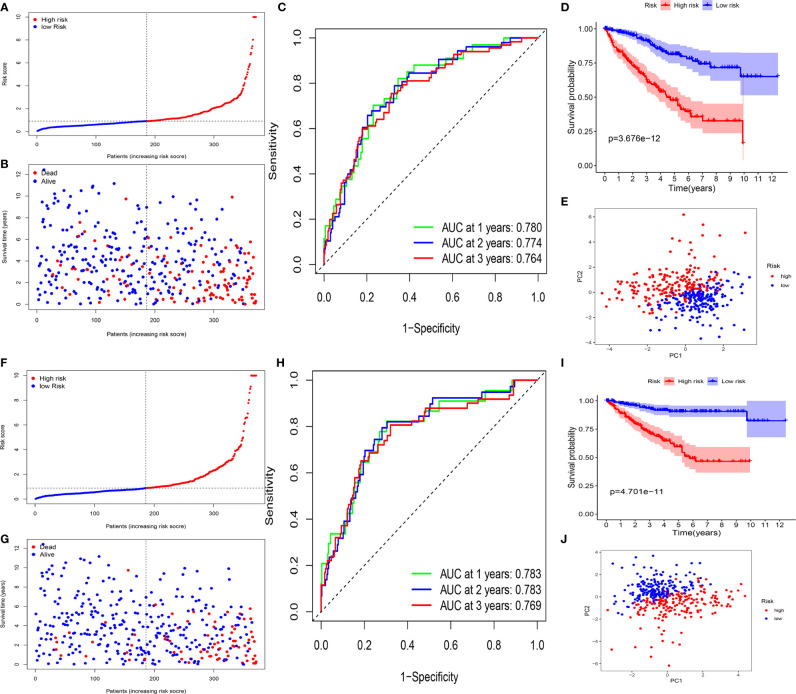
Survival analyses of the signature in the training cohort. **(A–E)** Survival analyses for patients with OS. **(A)** Risk score distribution of patients. **(B)** Survival status scatter plot. **(C)** Time-dependent ROC curve. **(D)** Kaplan–Meier curve. **(E)** PCA plot based on the risk score. **(F–J)**. Survival analyses for patients with CSS. **(F)** Risk score distribution of patients. **(G)** Survival status scatter plot. **(H)** Time-dependent ROC curve. **(I)** Kaplan–Meier curve. **(J)** PCA plot based on the risk score.

The ccRCC patients were categorized into a high-risk group (n = 185) and a low-risk group (n = 186) (**Figure 5A**). Kaplan–Meier curve indicated that high-risk patients had a significantly worse OS compared with the low-risk group patients (P <0.001) (**Figure 5D**). Time-dependent ROC curves were applied to evaluate the predictive capability of the risk score for OS. All the AUC values reached 0.70 (**Figure 5C**). The survival status scatter plot showed that the ccRCC patients classified as the high-risk group had a poor prognosis than those classified as low-risk (**Figure 5B**). The principal component analysis (PCA) plot indicated that the patients in different risk groups were distributed in two directions (**Figure 5E**). Moreover, cancer specific survival (CSS) analysis was performed. The patients in the training cohort were categorized into high- and low-risk groups (**Figure 5F**). The findings of CSS analysis were similar to the previous findings of OS (**Figures 5G–J**).

### Survival Analyses of the Eight Ferroptosis-Related Genes Prognostic Risk Signature in the Validation Cohorts

**Figure 6:**
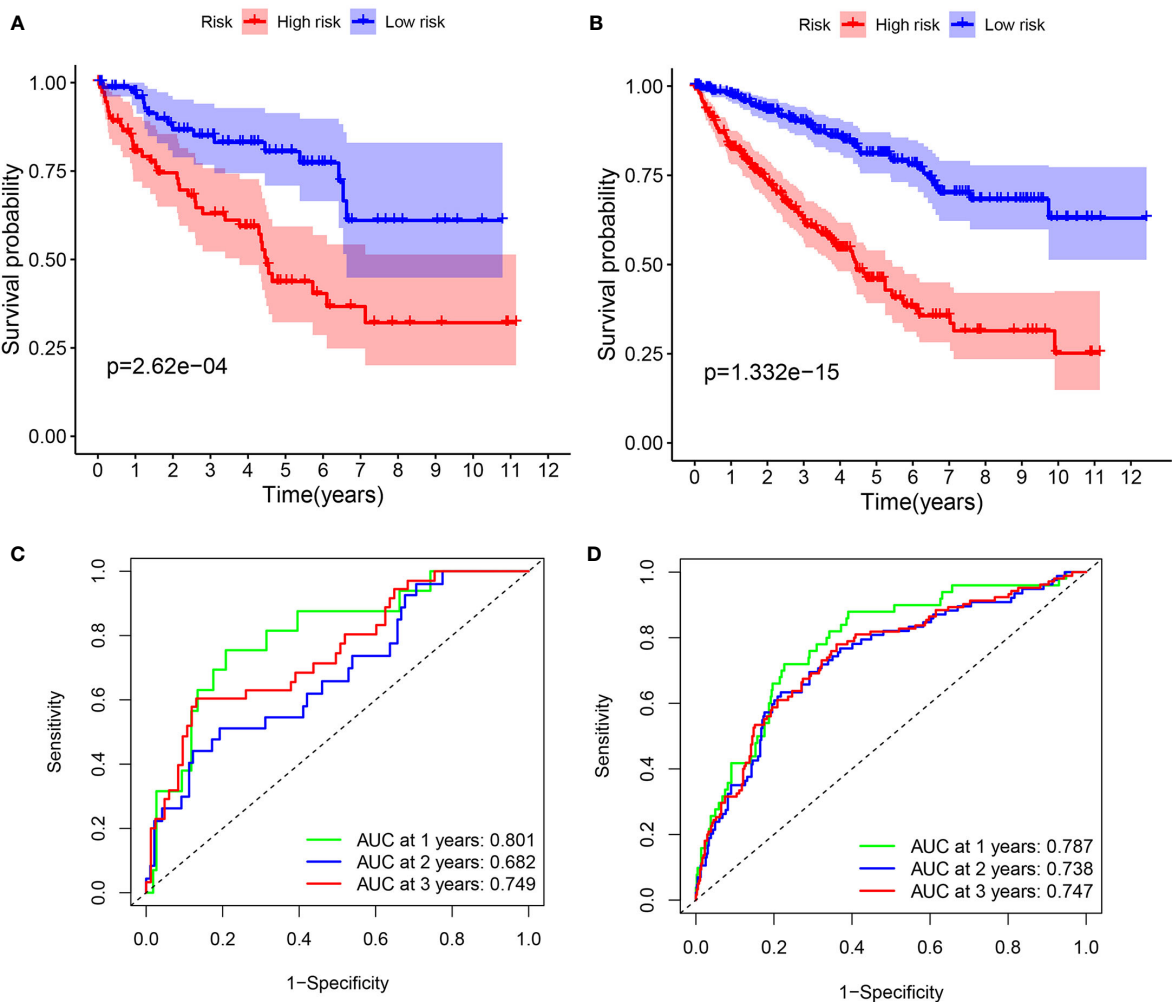
Survival analyses in the validation cohorts. **(A, B)** Kaplan–Meier curve of the testing cohort **(A)** and the entire TCGA cohort **(B)**. **(C, D)** Time-dependent ROC curve of the testing cohort **(C)** and the entire TCGA cohort **(D)**.

**Figure 7:**
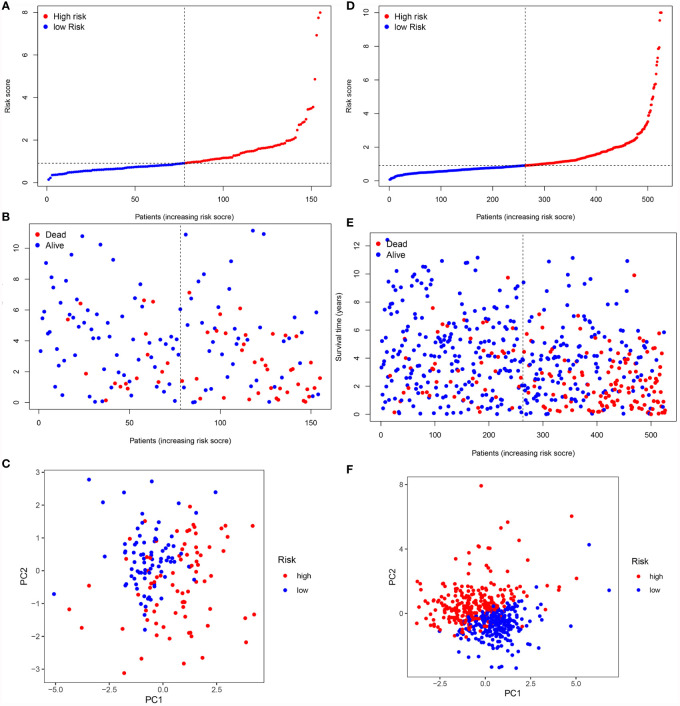
Validation of the signature in the validation cohorts. **(A, D)** Risk score distribution of patients in the testing cohort **(A)** and the entire TCGA cohort **(D)**. **(B, E)**. Survival status scatter plot of patients in the testing cohort **(B)** and the entire TCGA cohort **(E)**. **(C, F)** PCA plot in the testing cohort **(C)** and the entire TCGA cohort **(F)**.

To validate it, the risk score was also calculated in the testing cohort (n = 155) and the entire TCGA cohort (n = 576). In the testing cohort, 77 patients were classified as high-risk and 78 as low-risk, respectively (**Figure 7A**). Likewise, in the entire TCGA cohort, 263 patients were classified as high-risk and 263 patients as low-risk, respectively (**Figure 7D**). In line with the training cohort, lower OS could be found in patients with high-risk in both the testing cohort and the entire TCGA cohort (*P <*0.05) (**Figures 6A, B**). Next, the AUC of the 8-FRG risk model in the testing cohort was 0.801 in the 1st year, 0.682 in the 2nd year, and 0.749 in the 3rd year (**Figure 6C**). Accordingly, in the entire TCGA cohort, the AUC was 0.787 in the 1st year, 0.738 in the 2nd year, and 0.747 in the 3rd year (**Figure 6D**). All these ROC data were in line with the results of the training cohort. In addition, both the survival status scatter and PCA plots were shown in **Figures 7B, C, E, F**, respectively. All these data suggest that our model may contribute to the prognosis prediction of ccRCC patients.

### Identification of the Independent Prognostic Factors

**Figure 8:**
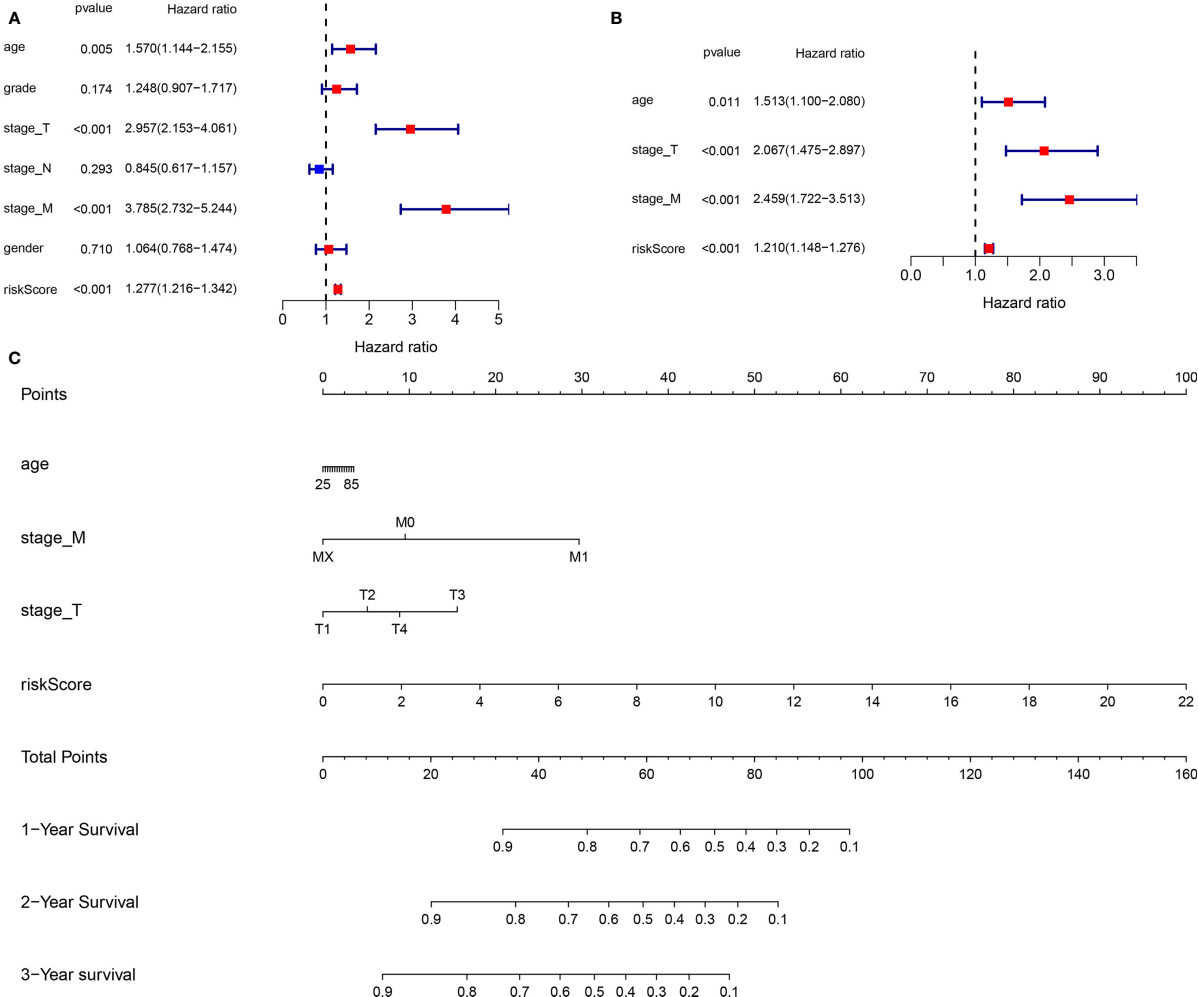
Construction of a new prognostic nomogram. **(A)** Univariate Cox regression analyses **(B)** Multivariate Cox regression analyses **(C)** Nomogram analyses of the selected prognostic factors.

In the entire TCGA cohort, the univariate and multivariate Cox regression analyses were performed to identify the independent prognostic factors among the risk score and clinical parameters (age, gender, grade, stage T, stages N and M). The univariate analysis indicated that clinical parameters (age, stages T and M) and risk score were correlated with ccRCC prognosis (*P <*0.05) (**Figure 8A**). The multivariate Cox regression analysis revealed that the risk score was independently associated with OS (*P <*0.05) (**Figure 8B**). Moreover, clinical variables such as age, stages T and M were also identified as the independent prognostic factors (*P <*0.05).

### Generation and Validation of a New Prognostic Nomogram

**Figure 9:**
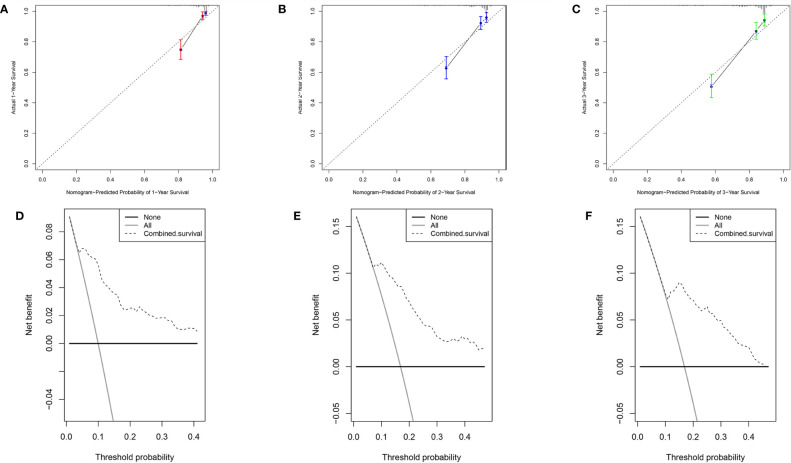
Verification of the nomogram performance. **(A)** The calibrate curve of 1st year. **(B)** The calibrate curve of 2nd year. **(C)** The calibrate curve of 3rd year. **(D)** The DCA plot of 1st year. **(E)** The DCA plot of 2nd year. **(F)** The DCA plot of 3rd year.

Base on the clinical features (age, stage T and stage M) and the risk score, a new prognostic nomogram was constructed to further predict ccRCC prognosis (**Figure 8C**). As validated by the calibrate curves and DCA curves, the nomogram had a favorable prognostic effect (**Figures 9A–F**).

### Risk Score of the Eight Ferroptosis-Related Genes Signature Had a Significant Correlation With the Immune Infiltration

**Figure 10:**
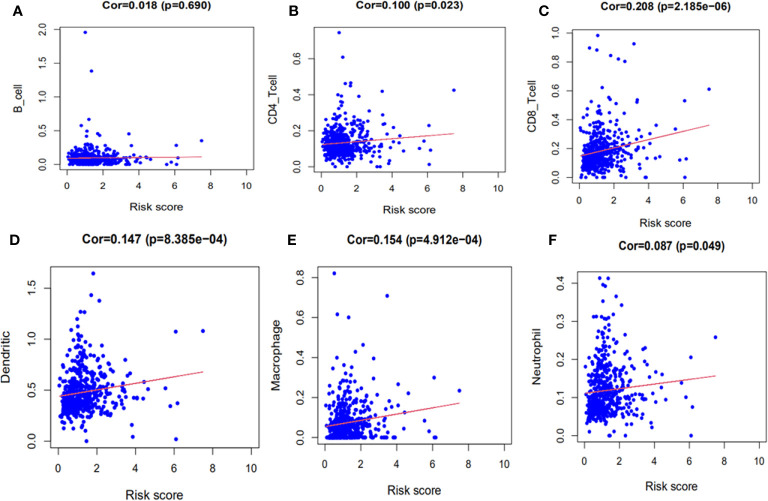
The immune correlation analyses of the signature. **(A)** B cells. **(B)** CD4+ T cells. **(C)** CD8+ T cells. **(D)** Dendritic cells. **(E)** Macrophages. **(F)** Neutrophils.

**Figure 11:**
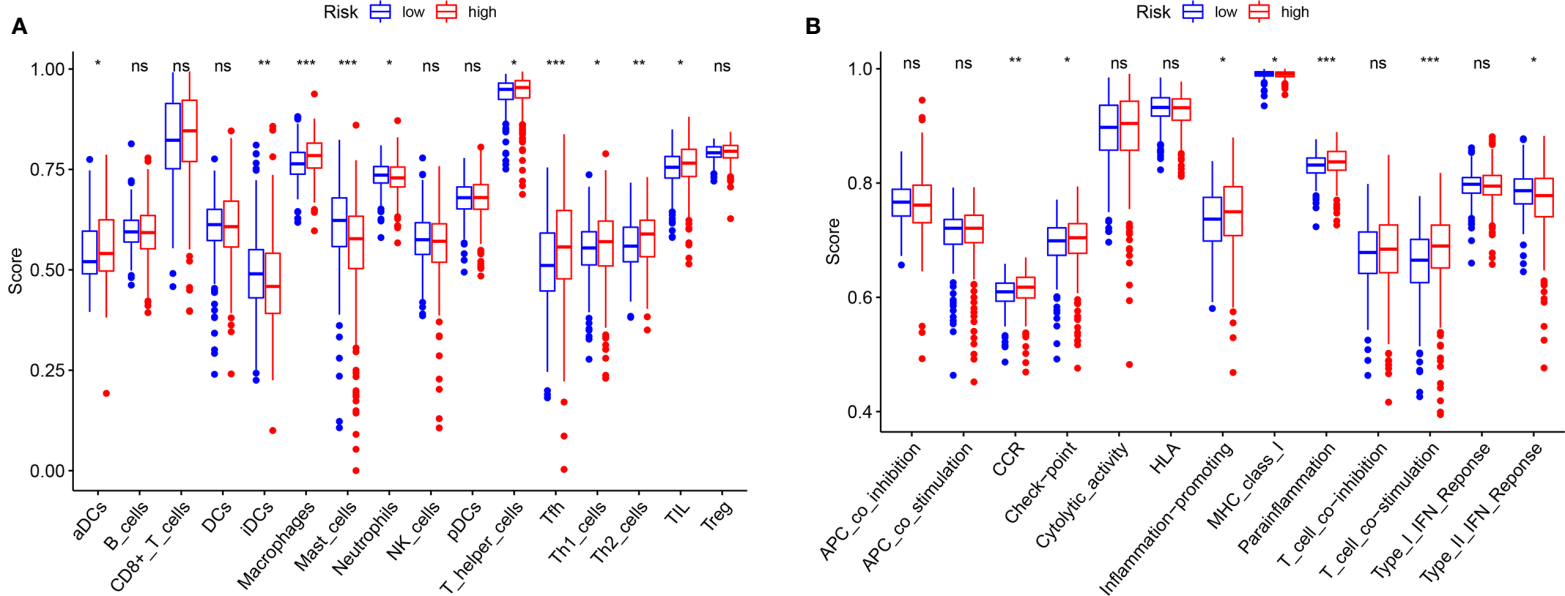
Comparison of the ssGSEA scores. **(A)** The scores of 16 immune cells are displayed in boxplots. **(B)** The scores of 13 immune-related functions are displayed in boxplots. Adjusted *P* values were showed as: ns, not significant; *P < 0.05; **P < 0.01; ***P < 0.001.

The immune correlation analysis revealed that this signature had a significant correlation with the levels of certain immune cells (CD4_T cell, CD8_T cell, neutrophils, macrophages and dendritic cells) in ccRCC (*P <*0.05) (**Figures 10A–F**). As per the enrichment scores based on the ssGSEA analyses, the levels of several immune cells, including the score of aDCs, iDCs, macrophages, mast_cells, Neutrophils, T helper_cells, Tfh, Th1_cells, Th2_cells, and TIL were significantly different between the different risk groups (*P <*0.05, **Figure 11A**). Immune pathway analysis showed that the score of type II IFN response had a negative association with the risk score of patients, while the T_cell_co-stimulation and parainflammation had the opposite effect (*P <*0.05, **Figure 11B**). Our results suggest that the signature significantly correlates with immune infiltration.

### Overall Survival Validation of Different Clinical Subgroups by Stratified Survival Analysis

**Figure 12:**
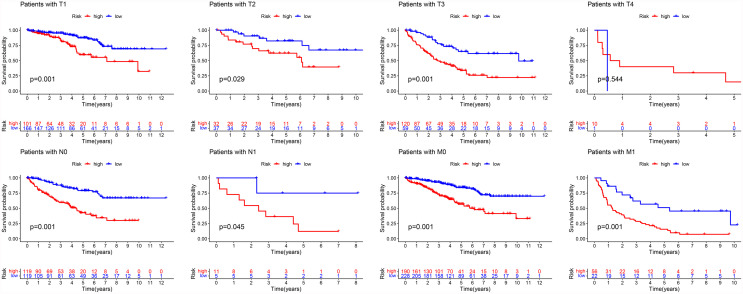
Stratified survival analysis of patients with different TMN stage.

The K–M survival curves indicated that in most subgroups categorized based on the TMN stage, the OS of low-risk patients was significantly better than the OS of those with high-risk (**Figure 12**, *P <*0.05). Only these patients with T4 stage were not eligible, which may be related to the low number of samples.

### Validation of the Expression of Ferroptosis-Related Genes in ccRCC

**Figure 13:**
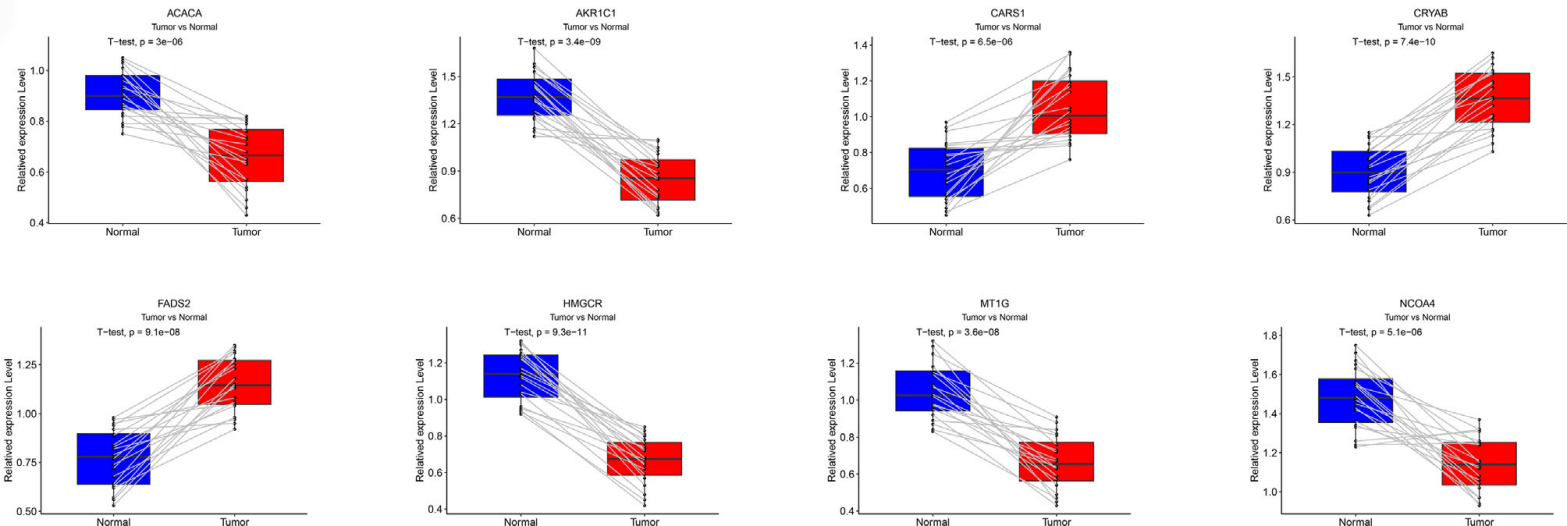
Validation of qRT-PCR. The mRNA expression levels for each FRG in the signature was verified by qRT-PCR.

qRT-PCR was performed to examine the mRNA expression levels of eight FRGs in 20 paired ccRCC and adjacent non-tumorous tissues. We found increased CARS1, CRYAB and FADS2 expression in ccRCC tissues as compared with adjacent non-tumorous tissues, while the expression of other five FRGs was reduced in ccRCC (**Figure 13**).

## Discussion

With the increase in the novel treatment options for ccRCC, promising biomarkers for monitoring the ccRCC prognosis are urgently needed (22). Disorders of FRGs have been reported in numerous malignant tumors, suggesting a vital role of FRGs in tumor progression (23, 24). The abnormal FRGs are reported to be involved in the initiation and progression of ccRCC (12, 25). However, the comprehensive understanding of FRGs and ccRCC prognosis remains largely unknown.

The results of the present study established a novel ferroptosis-related prognostic gene signature for ccRCC patients. We systemically explored the prognosis and function of significant FRGs and identified 26 PDEFRGs in ccRCC. Further, we constructed the signature of eight FRGs. This signature contributed to a better prediction of ccRCC prognosis and provided potential therapeutic targets for ccRCC.

AKR1C1, CARS1, HMGCR, CRYAB, MT1G, NCOA4, ACACA and FADS2 are the FGRs included in the eight-FRG signature. AKR1C1 plays a key role in the regulation of autophagy and oxidative stress in the non-small cell lung cancer (26). Down regulation of ACACA expression is associated with the inhibition of malignant progression of prostate cancer (27). Nie et al. constructed a novel prognostic signature involving CARS1, which effectively predicted the prognosis of colon cancer (28). CRYAB has been reported to be a potential therapeutic target for nasopharyngeal carcinoma (29). The inhibition of HMGCR stabilizes the glycolytic enzyme PKM2 and promotes the growth of RCC (30). MT1G is reported to be hypermethylated in RCC (31). Low expression of NCOA4 is associated with ccRCC progression, and poor prognosis and immune infiltration in ccRCC patients (12). Wu et al. developed an 11 metabolic gene signature-based prognostic model in ccRCC (32). Interestingly, FADS2, which is incorporated in our model, was also incorporated in their model. But, only HMGCR and NCOA4 were explored in RCC, whereas the other six FRGs were not investigated. Herein, we examined the expression of eight FRGs using qRT-PCR in paired ccRCC and adjacent non-tumorous tissues.

Recently, lines of evidence have demonstrated that the immune infiltration participates in the progression of ccRCC. For instance, Chakiryan et al. found that common somatic mutations in ccRCC may correlate with immune infiltration (33). Bai et al. also found that various types of immune cells and the immune functions are correlated with ccRCC progression (34). It is known that ferroptosis could trigger dendritic cell maturation to exert their anti-tumor immune effects (35). T-cells play an important role in the tumor topology and efficacy of various therapeutic strategies for ccRCC (36). In addition, ccRCC with high expression of C4-activating enzyme C1s, may involve the infiltration of macrophages and T cells (37). Therefore, whether the risk score of our prognosis model is associated with immune cell infiltration was explored. Interestingly, with the increase in risk score, the levels of immune cells (CD4_T cell, CD8_T cell, neutrophils, macrophages and dendritic cells) were also increased. Our data suggest that the signature of eight FRGs is associated with immune cell infiltration.

Recently, the prognosis prediction potential of FRGs has been explored in many human cancers. For example, Zhu et al. demonstrated the utility of a 4-FRGs model in predicting the prognosis of esophageal adenocarcinoma (38). Zheng et al. developed a 12-FRGs model to better predict the prognosis of patients with lower-grade gliomas (39). Jiang and his colleagues constructed an eight-gene ferroptosis-related prognostic model to predict the prognosis of gastric cancer patients (40). Our study has many advantages. Firstly, a novel 8-FRG prognostic risk signature for ccRCC was constructed, which contributes to the ccRCC prognosis prediction. Secondly, clinical features are integrated into the 8-FRG model to construct a nomogram, which improves the prognosis prediction ability in ccRCC. Finally, this signature is significantly correlated with immune cell infiltration. More clinical databases should be used to verify the accuracy of this 8-FRG prognostic risk signature in the future studies.

In conclusion, we disclose a novel 8-FRG prognostic risk signature for ccRCC, contributing to the prognosis prediction of ccRCC patients.

## Data Availability Statement

Publicly available datasets were analyzed in this study. This data can be found here: https://portal.gdc.cancer.gov/ and http://cistrome.org/.

## Ethics Statement

The studies involving human participants were reviewed and approved by the Human Research Ethics Committee in The First Affiliated Hospital of Wenzhou Medical University. The patients/participants provided their written informed consent to participate in this study. Written informed consent was obtained from the individual(s) for the publication of any potentially identifiable images or data included in this article.

## Author Contributions

JC and YL designed the study and analyzed the data. YZ, RZ, BC and JH revised the images. WZ, YW and YG performed the literature search and collected data for the manuscript. CL and JZ revised the manuscript. All authors contributed to the article and approved the submitted version.

## Funding

The project was supported by the National Natural Science Foundation of China (No. 81873576), the Medical Health Science and Technology Project of Zhejiang Provincial Health Commission (No. 2020RC081) and the project of Wenzhou Medical University Basic Scientific Research (No. KYYW201904).

## Conflict of Interest

The authors declare that the research was conducted in the absence of any commercial or financial relationships that could be construed as a potential conflict of interest.

## Abbreviations

RCC, renal cell carcinoma; ccRCC, clear cell renal cell carcinoma; TCGA, The Cancer Genome Atlas; FRGs, ferroptosis-related genes; Lasso, Least absolute shrinkage and selection operator; DEFRGs, differentially expressed ferroptosis-related genes; PDEFRGs, prognostic differentially expressed ferroptosis-related genes; TFs, transcription factors; FDR, false-discovery rate; OS, overall survival; AUC, area under the curve; ROC, receiver operating characteristic; ssGSEA, Single-sample gene set enrichment analysis; PCA, Principal component analysis; Tfh, T follicular helper cell; TIL, Tumor Infiltrating Lymphocyte; aDC, Activated dendritic cell; iDC, Immature dendritic cell; PPI, protein–protein interaction; CSS, cancer specific survival; qRT-PCR, Quantitative Real-time PCR.
